# Application of Methods for a Morphological Analysis of the Femoral Diaphysis Based on Clinical CT Images to Prehistoric Human Bone: Comparison of Modern Japanese and Jomon Populations from Hegi Cave, Oita, Japan

**DOI:** 10.1155/2022/2069063

**Published:** 2022-06-07

**Authors:** Daisuke Endo, Kazunobu Saiki, Minoru Yoneda, Hajime Ishida, Keiko Ogami-Takamura, Rina Sakai, Kiyohito Murai, Takeshi Imamura, Yoshiatsu Naito, Tetsuaki Wakebe, Toshiyuki Tsurumoto

**Affiliations:** ^1^Department of Macroscopic Anatomy, Graduate School of Biomedical Sciences, Nagasaki University, 1-12-4 Sakamoto, Nagasaki, Nagasaki 852-8523, Japan; ^2^The University Museum, The University of Tokyo, 7-3-1 Hongo, Bunkyo-ku, Tokyo 113-0033, Japan; ^3^Department of Human Biology and Anatomy, Graduate School of Medicine, University of the Ryukyus, Senbaru 1, Nishihara, Okinawa 903-0213, Japan; ^4^Center of Cadaver Surgical Training, School of Medicine, Nagasaki University, 1-12-4 Sakamoto, Nagasaki, Nagasaki 852-8523, Japan; ^5^Department of Anesthesiology and Intensive Care Medicine, Graduate School of Biomedical Sciences, Nagasaki University, 1-7-1 Sakamoto, Nagasaki, Nagasaki 852-8501, Japan; ^6^School of Medicine, Nagasaki University, 1-12-4 Sakamoto, Nagasaki, Nagasaki 852-8523, Japan; ^7^Nagasaki University, 1-12-4 Sakamoto, Nagasaki, Nagasaki 852-8523, Japan; ^8^Nagasaki Medical College, 36-59 Atago, Nagasaki, Nagasaki 850-0822, Japan

## Abstract

A morphological analysis of ancient human bones is essential for understanding life history, medical history, and genetic characteristics. In addition to external measurements, a three-dimensional structural analysis using CT will provide more detailed information. The present study examined adult male human skeletons excavated from Hegi cave, Nakatsu city, Oita Prefecture. CT images were taken from the femurs of adult males (Initial/Early Jomon Period (*n* = 10) and Late Jomon Period (*n* = 5)). Cross-sectional images of the diaphysis from below the lesser trochanter to above the adductor tubercle were obtained using the method established by Imamura et al. (2019) and Imamura et al. (2021). Using Excel formulas and macros, the area of cortical bone, thickness, and degree of curvature were quantitatively analyzed. The results were compared with data on modern Japanese. The maximum thickness of cortical bone in the diaphysis and the degree of the anterior curvature were significantly greater in Late Jomon humans than in the other groups. In contrast to modern humans, the majority of Jomon femurs showed the S-shaped curvature with the medial side at the top position and the lateral side at the lower position. The present results demonstrate that Late Jomon humans had a wider range of activity than the other groups and also provide insights into diseases in the hip and knee joints of Jomon humans.

## 1. Introduction

A morphological analysis of bone provides various types of information, such as the risk of fracture. Relationships have been reported between the angle between the femoral head and diaphysis [1], the increased curvature of the diaphysis, and atypical fractures in the femur [2–4]. Furthermore, as the lateral curvature of the femur increases, fracture sites have been shown to move from below the subtrochanteric region to the midportion of the diaphysis [5, 6]. On the other hand, the removal of cancellous bone from the femoral neck did not significantly change the strength of the femur [7], suggesting that cortical bone defines the strength of the femur. The risk of femoral fracture is higher in women, and even after standardization for body size, the femoral diaphysis is still thinner in women than in men and possesses less cortical bone [8]. Therefore, the morphology of cortical bone in the femoral diaphysis may be important for predicting the risk of fracture; however, a simple and reproducible method for its assessment has not yet been established. Although the increased curvature of the diaphysis has been implicated as a risk factor for atypical fractures, there is currently no unified method for a quantitative analysis of anterior and lateral curvatures with high reproducibility. Therefore, we developed methods to quantitatively analyze the morphology of cortical bone using CT images of modern Japanese skeletal specimens, with a threshold value that distinguishes cortical bone from cancellous bone [9, 10]. This method may become the standard for a morphological analysis because it is completed in Excel and does not require specific software. Using this method, it is possible to obtain quantitative values for the periosteal border length (PBL), the area of cortical bone in a cross-section, cortical bone occupancy (cortical index (CI)) relative to the total area of the cross-section, cortical bone thickness, and the degrees of the anterior and lateral curvatures of the diaphysis relative to the bone axis. This analysis revealed marked diversity in the morphology of cortical bone and also showed that the area of cortical bone, CI, and cortical bone thickness were affected by sex and age. We previously demonstrated that bone morphology correlated with mechanical functions by comparing these quantitative values to the findings of a finite element analysis using a 3D model constructed from CT images [11]. In addition to the estimation of disease risk, bone morphology has been considered to reflect the living environment of adults [12–14] based on Wolff's law that bone morphology changes to adapt to a given load [15]. Therefore, differences in the curvature of the femoral diaphysis among populations and differences in femoral morphology among various groups of archaic human bones have been analyzed to investigate the living environment [16, 17]. Novel insights are anticipated with the application of the methods already established for a morphological analysis to archaic human bones. Therefore, the present study examined femurs excavated from Hegi cave. Sixty-seven ancient human bones have been excavated from Hegi cave in Nakatsu city, Oita Prefecture, Kyushu Island, Japan [18]. Fifteen male femurs with well-preserved diaphyses were selected for analyses. There are two reasons why we chose males. The first is that we were able to analyze a statistically processable number of male femurs from different periods of the Jomon period. The other reason is that the age at death of human bones excavated from the Hegi cave cannot be strictly estimated, and the bones used for analysis can only be said to be those of adults. The results of the analysis by Imamura et al. showed that age had a smaller effect on morphological parameters in males than in females, and no significant correlation has been found [9]. Therefore, the influence of age was considered small in comparison with modern humans. These bones were dated using the ^14^C method established by Yoneda et al. [19] and divided into two groups: those belonging to the Initial/Early Jomon Period and to the Late Jomon Period. We compared measurements in each group with those of modern humans [7, 8]. The Late Holocene is dated to the Late Jomon Period, while the other groups are roughly dated to the Initial/Early Jomon Period. In the Holocene, which began with the end of the Ice Age, the Late Holocene is the period during which temperatures dropped and many settlements disappeared [20, 21]. Changes in the living environment may be reflected not only by comparisons between modern humans and the prehistoric remains excavated from Hegi cave but also differences in the ages of the prehistoric remains.

## 2. Materials and Methods

**Figure 1:**
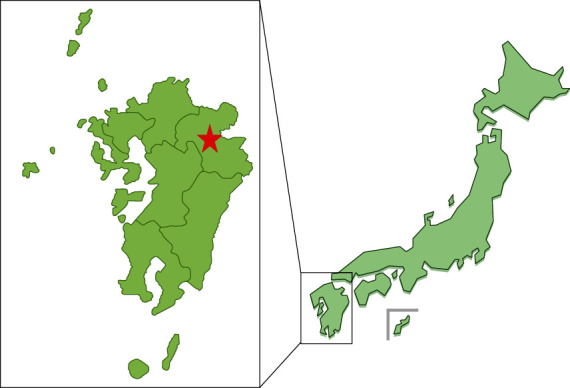
Location of Hegi cave in Nakatsu city, Oita Prefecture, Japan.

Human femurs were excavated from Hegi cave in Nakatsu city, Oita Prefecture, Japan (Figure 1). The skeletal remains from the cave have been legally preserved in the Department of Macroscopic Anatomy, Graduate School of Biomedical Sciences, Nagasaki University, which was involved in the excavation project [18].

### 2.1. Radiocarbon Dating of Samples

**Figure 2:**
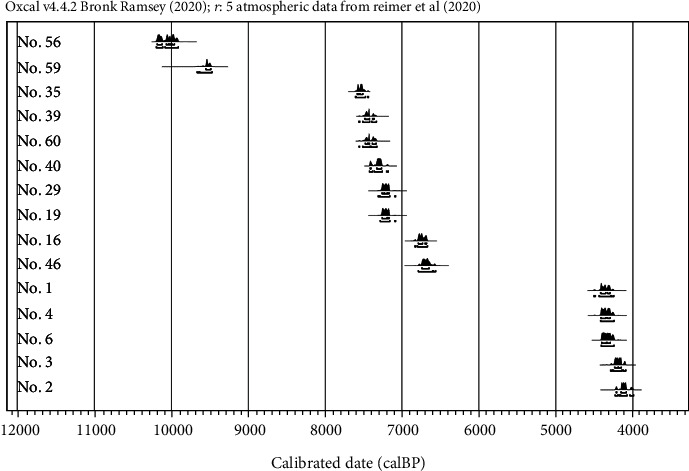
Probability distributions of calibrated ^14^C dates of the femurs excavated from Hegi cave.

The methods used to date the bones analyzed were previously reported by Yoneda et al. [19]. In brief, gelatine from biogenic collagen was extracted from bones. For the ^14^C analysis, 1.2–2.5 mg of collagen, containing approximately 0.5–1 mg of carbon, was oxidized to CO_2_ in evacuated tubes with copper dioxide at a temperature of 850°C, and CO_2_ was then cryogenically purified in a vacuum system [22]. The radiocarbon content of the mixture of graphite and iron powder was measured using accelerator mass spectrometry (AMS) at The University of Tokyo [23]. Fifteen femurs were divided into the Initial/Early Jomon (*n* = 10, ca. 10,200-6,500 cal BP) and Late Jomon (*n* = 5, ca. 4,500-4,000 cal BP) groups based on the results of radiocarbon dating (Table 1 and Figure 2).

### 2.2. Assessment of Age and Sex

The age and sex of the Jomon paleo-human remains analyzed were established based on the morphological features of the skull and pelvic bone and reported in Chapter 6 of the Honyabakei Town History [18]. Fifteen male femurs in good condition were selected from 67 archaeological remains. The ages of the owners of those femurs were all estimated to be adults over 20 years old.

### 2.3. CT Imaging and Extraction of Target Images

**Figure 3:**
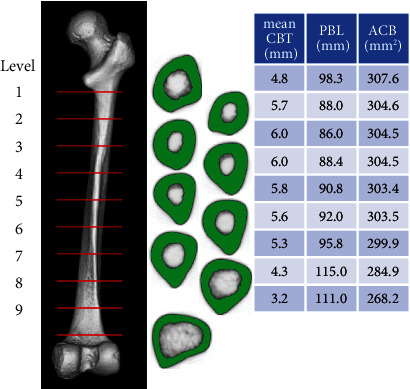
Example of results from the Late Jomon Period population. The shapes of cross-sections at eight levels are shown. In the table, the values for mean cortical bone thickness (mean CBT), periosteal border length (PBL), and the area of cortical bone (ACB) are shown. The image was composed with CT images using Radiant DICOM viewer (ver 2020 1.1 (64-bit), Mexidant, Poznan, Poland).

Full-length images of all examined femurs were obtained using clinical multislice CT (Activision 16, Toshiba Corp., Tokyo, Japan) (X − tube volume/current = 120 kV/100 mA, image matrix size: 512 × 512 pixels, and slice thickness: 0.5 mm) installed at the Graduate School of Biomedical Sciences, Nagasaki University. This slice thickness resulted in an error of up to 0.6% in morphological parameters. Since the plane resolutions of CT were assessed using a field size divided by 512, they were not constant and ranged between 0.158 and 0.292 mm (mean value, 0.213 mm). Bones were placed with the posterior side down and grounded on the table of the imaging device at three points: the most posterior points of the greater trochanter, medial condyle, and lateral condyle. Data were saved in the Digital Imaging and Communication in Medicine (DICOM) format. The range between the lower end of the lesser trochanter and adductor tubercle of each femur was divided into nine segments of equal lengths. Cross-sections, including both ends, were labeled from the top to bottom as “level 1” to “level 10” (Figure 3). Threshold values for the definition of the cortex were calculated as described in our previous study [9] and assessed as follows: (i) all of the matrixes for the ten levels were pasted into one Microsoft Excel sheet, and (ii) a histogram was created based on a frequency table of Hounsfield units (CT values) to calculate the mean CT value for the first peak (i.e., approximately -1000; mainly indicating the CT value of the surrounding air) and the CT value for the second peak (i.e., indicating the CT value of the bone itself).

### 2.4. Quantification of Morphological Elements of the Femoral Diaphysis Using an Image Analysis

The methods used to quantify the morphological parameters of cross-sections of the femoral diaphysis using CT data were previously reported by Imamura et al. [9]. All calculations were conducted using Microsoft Excel. In brief, cross-sectional areas were calculated by counting all points surrounded by the periosteal perimeter. Regarding the area of cortical bone, all points surrounded by both the periosteal and endosteal perimeters of cortical bone were counted. This area was corrected by calculating the actual length per pixel in DICOM data to consider the magnification ratio at the time of imaging. The ratio of the area of cortical bone to the cross-sectional area was calculated as the cortical index (CI). PBL in each section was calculated by counting the number of all points on the periosteal surface. To evaluate cortical bone thickness, distances between a point on the periosteal surface and all points on the endosteal surface of cortical bone were calculated. The minimum of these values was defined as the cortical bone thickness of the point. Calculations were performed for all points of the periosteal surface of cortical bone. The maximum and mean values of all points were defined as maximum and mean cortical bone thicknesses, respectively. The measurement results were represented without and with standardization by dividing by the total length of each femur.

### 2.5. Quantification of the Degree of Curvature by an Image Analysis

The method used to quantify the degree of curvature was reported by Imamura et al. [10]. The central mass distribution (CMD) of cross-sectional images was assessed as the intersection of two line segments dividing any two-dimensional figure into equal areas. The CMD curve was obtained by connecting all CMDs of the nine cross-sections of the femur. Shifts in the *x*- and *y*-axis directions of CMD at each level from the line connecting the CMDs of the first and ninth cross-sections were calculated.

### 2.6. Statistical Analysis

The goodness-of-fit test was performed to confirm the standard distribution of each parameter. A repeated measures two-factor ANOVA followed by multiple comparisons with Scheffé's test was conducted to examine differences in morphological parameters among the modern, Initial/Early Jomon, and Late Jomon populations. A principal component analysis was performed using the mean value of each morphological element. Regarding the lateral curvature, we investigated whether the distribution of each curvature pattern differed among the modern, Initial/Early Jomon, and Late Jomon populations using the chi-squared independence test. Statistical analyses were conducted with Excel-based formulas and macros.

## 3. Results

### 3.1. A New Analysis Method Based on CT Images Revealed Differences in the Femoral Diaphysis between Modern and Jomon Humans

**Figure 4:**
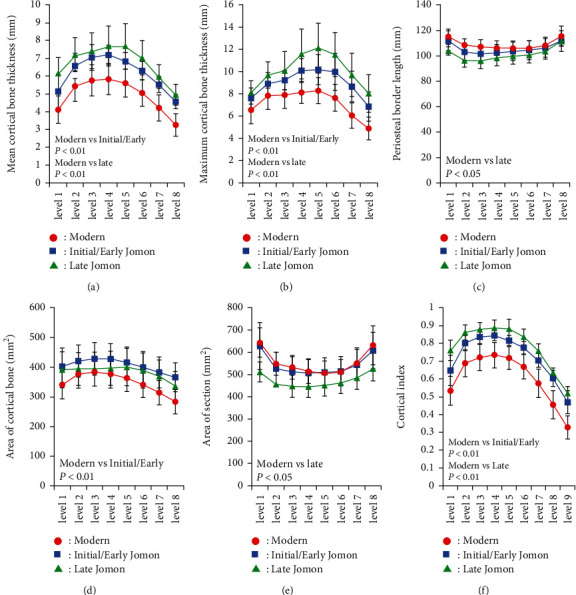
Morphological parameters of modern (Imamura, et al., [9]), Initial/Early Jomon, and Late Jomon Period populations. (a) Mean cortical bone thickness, (b) maximum cortical bone thickness, (c) periosteal border length, (d) area of cortical bone, (e) area of sections, and (f) cortical index. Averages are plotted at each cross-sectional level. Error bars indicate ±1 standard deviation. Significant differences analyzed by a two-way ANOVA followed by multiple comparisons were indicated in each graph.

**Figure 5:**
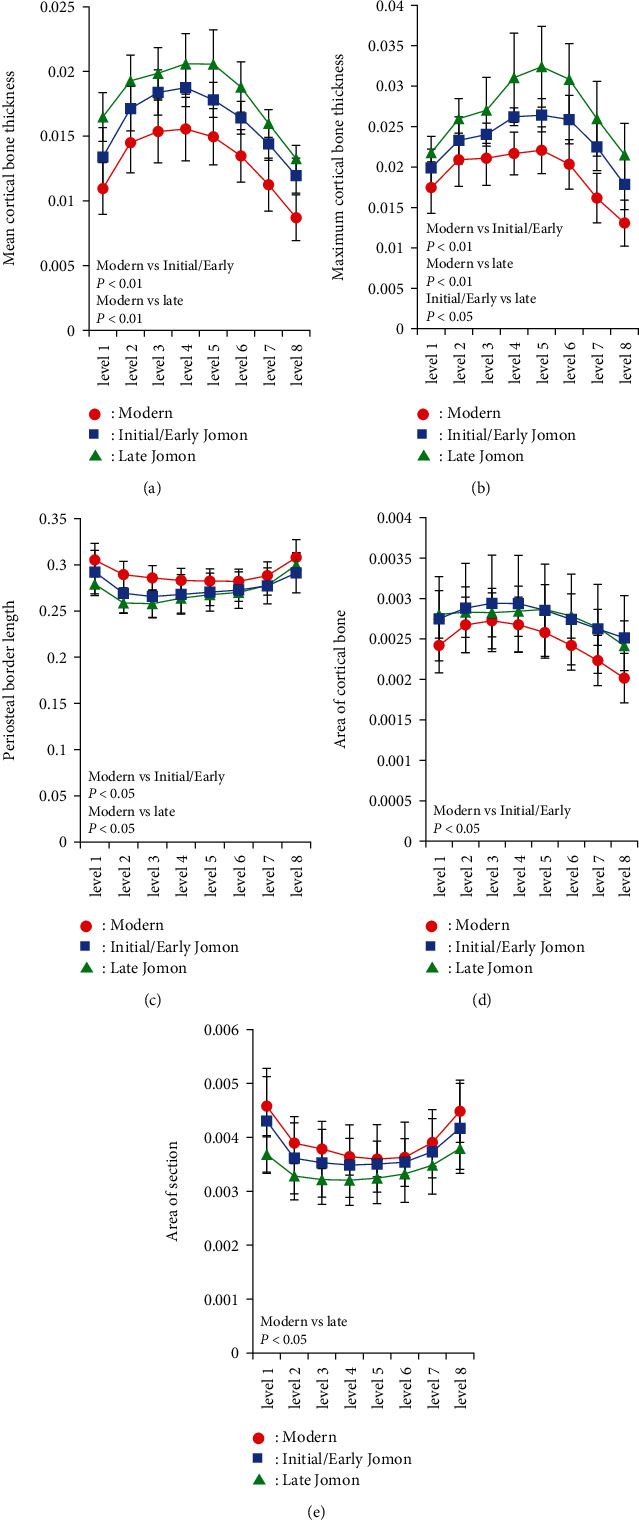
Morphological parameters standardized with diaphyseal lengths of modern (Imamura, et al.,[9]), Initial/Early Jomon, and Late Jomon Period populations. (a) Mean cortical bone thickness, (b) maximum cortical bone thickness, (c) periosteal border length, (d) area of cortical bone, and (e) area of section. Averages are plotted at each cross-sectional level. Error bars indicate ±1 standard deviation. Significant differences analyzed by a two-way ANOVA followed by multiple comparisons were indicated in each graph.

**Figure 6:**
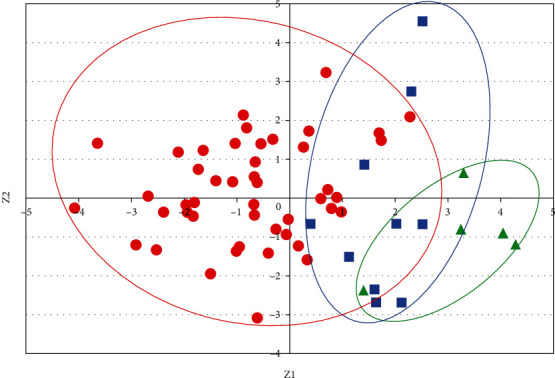
Principal component analysis of means of morphological parameters standardized with diaphyseal lengths. Red circles, blue squares, and green triangles indicate the principal component scores of modern, Initial/Early Jomon, and Late Jomon Period populations, respectively. Eigenvectors for each score, *Z*1 and *Z*2, are shown in Table 2.

Mean and maximum cortical bone thicknesses, PBL, the area of cortical bone, the total cross-sectional area, and CI were measured in 8 cross-sections of the femoral diaphysis (Figure 4). The mean, maximum, and CI of cortical bone thickness were higher at all levels in the order of the Late Jomon, Initial/Early Jomon, and modern populations. On the other hand, PBL and the total cross-sectional area were higher in the order of the modern, Initial/Early Jomon, and Late Jomon populations. Similar results were obtained when measurements were standardized by the length of the femoral diaphysis to exclude the effect of height (Figure 5). A two-way analysis of variance showed more combinations of significant differences after standardization, particularly for maximum cortical bone thickness, not only between the Jomon and modern populations but also between the Initial/Early and Late Jomon populations. A principal component analysis was performed using the mean values at all levels for the five morphological factors standardized by femoral diaphyseal length and CI. Eigenvectors obtained for the first and second principal component scores are shown in Table 2. The eigenvector for the first principal component was positive, except for CI, which was considered to reflect the size of the cross-section because the contributions of the cross-sectional area and perimeter diameter were large. On the other hand, CI and cortical bone thickness contributed positively, while the cross-sectional area and PBL contributed negatively to second principal component scores, reflecting the size of cortical bone. A scatter plot with the first principal component score on the *x*-axis and the second principal component score on the *y*-axis showed groups of bones in the order of the modern, Initial/Early Jomon, and Late Jomon populations (Figure 6).

### 3.2. The Degree and Pattern of Curvature Differs between Modern and Jomon Humans

**Figure 7:**
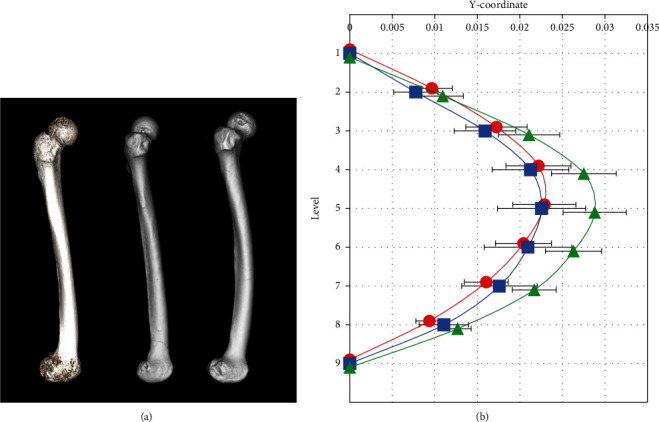
Comparison of the anterior curvature among modern, Initial/Early Jomon, and Late Jomon Period populations. (a) Examples of modern (left), Initial/Early Jomon (middle), and Late Jomon (right) Period populations. (b) Averaged degrees of the anterior curvature normalized with femoral lengths are shown. Circles represent the modern population, squares represent the Initial/Early Jomon Period population, and triangles represent the Late Jomon Period population. Averages are plotted at each cross-sectional level. Error bars indicate ±1 standard deviation. Significant differences were found between modern and Late Jomon Period populations (*p* < 0.01) and between the Initial/Early and Late Jomon Period populations (*p* < 0.05).

**Figure 8:**
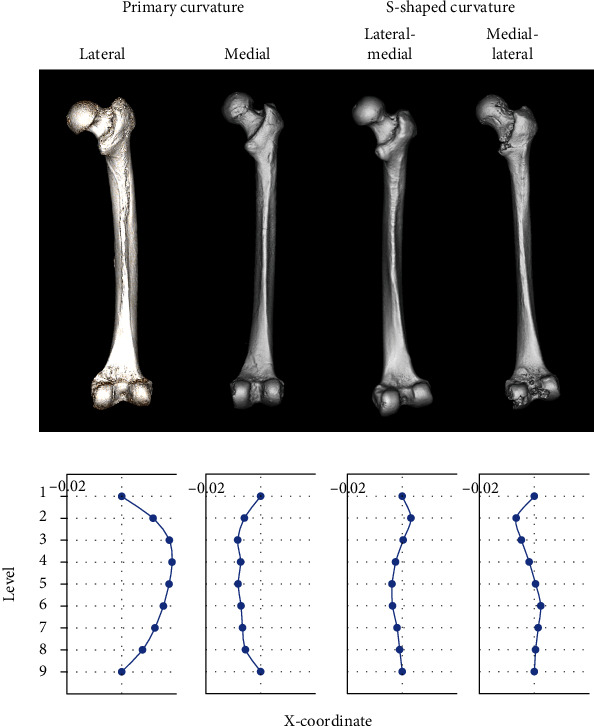
Examples of four types of the lateral curvature.

**Figure 9:**
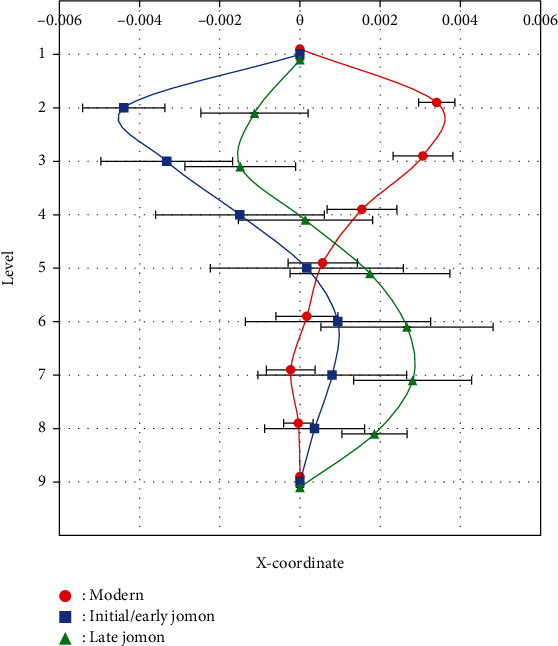
Averages of *x*-coordinates of modern, Initial/Early Jomon, and Late Jomon Period populations. Averages are plotted at each cross-sectional level. Error bars indicate ±1 standard error.

The curvature of Jomon femurs excavated from Hegi cave was quantified using the method established by Imamura et al. [10]. All of the Jomon femurs were flexed anteriorly, and in comparisons with modern bones, the degree of curvature of Initial/Early Jomon bones was similar to that of modern bones, while Late Jomon bones showed significantly stronger curvature than modern and Initial/Early Jomon bones (Figure 7). While the anterior curvature of both Jomon and modern bones showed a primary curve with the apex in the middle of the femoral diaphysis, the lateral curvature showed a more diverse pattern (Figure 8). The majority of modern bones showed a lateral primary curve with the apex at the top of the femur diaphysis. Some of the modern and Jomon bones also showed a primary curve of the medial curvature with an apex at the top of the diaphysis. On the other hand, many femurs had cross-sections with both medial and lateral center points relative to the bone axis connecting the center points of levels 1 and 9. In contrast to previous findings on modern bones, which all showed the lateral curvature at the top of the diaphysis and the medial curvature at the bottom, most of the Jomon bones showed the medial curvature at the top and the lateral curvature at the bottom, and the distribution of curvature patterns significantly differed among the groups (Table 3).  Figure 9 shows a graph with mean curvature values at each level. Mean values and standard errors were used because some bones showed different kyphotic patterns. In contrast to the modern human skeleton, which has the lateral curvature at the top of the diaphysis, the Jomon human skeleton has the medial curvature at the top and the lateral curvature at the bottom, with the medial curvature at the top being more pronounced in the Initial/Early Jomon human and the lateral curvature at the bottom being more pronounced in the Late Jomon human.

## 4. Discussion

In the present study, we quantitatively analyzed the morphological features of cross-sections of the femoral diaphysis of Jomon humans excavated from Hegi cave based on CT images using an already established method for skeletal specimens of the modern Japanese population (Imamura et al., [9]; Imamura et al. [10]). Among the male femurs excavated, 15 with well-preserved diaphyses were selected for analysis. The acquisition of CT images, extraction of cross-sections at each level, the setting of thresholds for the definition of cortical bone, and the acquisition of quantitative values were performed without any modifications from the protocol established for the modern Japanese population, indicating that the method is applicable to the measurement of archaic human bones and that the values obtained can be directly compared with those of the modern Japanese population. Femoral morphology is important not only for its clinical significance in predicting fracture risk [5–8] but also in terms of predicting the living environment [12–14]. However, as our previous studies have shown, the trends of age-related changes were different for diaphyseal peripheral border length and thickness of cortical bone. On the other hand, the thickness of cortical bone not peripheral border length was strongly correlated with the stress and strain applied to the femur with one-legged standing configuration estimated with the finite element method [11], suggesting that external measurement of femoral morphology alone is insufficient to estimate its function and that it is essential to analyze its internal structure. For this reason, many analyses have been performed using CT [24–26], but measurement conditions must be uniform to compare results among different populations. Various algorithms have been used to define cortical bone [27, 28], but no reproducible method has been established because it requires special software or programs and subjective judgment by the researcher. The method used in this study was completed on the highly generalized software Excel, and after CT images were acquired under the conditions described in this paper, the determination of threshold values to define cortical bone and the acquisition of analysis results could be performed mechanically, without requiring subjective judgment. No anomalous values that could be attributed to the method or results inconsistent with the impression received from the CT images were obtained during the analysis process. The fact that the method was applicable not only to modern bleached human bones but also to archaic femurs strongly suggests that the method can be applied to a very wide range of specimens.

Quantitative analysis of curvature was also possible by clearly separating anterior and lateral curvature. As discussed below, the anterior and lateral curvature is affected by different mechanical conditions, so this separation is essential to consider their background and significance. The degree of curvature of the diaphysis has been expected to be a distinguishing feature between different populations since Stewart's work in 1962 and has been studied not only about differences between Japanese and Ainu but also about behavior [17, 29, 30]. Since a unified method is still needed to facilitate such research, the importance of this method is very high. In the future, by estimating the results of biomechanical analysis from the multiple morphological data, it will be possible to compare femoral functions among different populations. Jomon Period femurs excavated from Hegi cave appeared to be thinner than modern femurs, while no significant differences were observed in femur lengths. This result was confirmed by a morphological analysis based on CT images. PBL of the diaphysis was the largest in modern femurs, and when standardized by the length of the diaphysis, modern femurs were significantly thicker than those from both the Initial/Early and Late Jomon Periods. In addition, the cross-sectional area of the modern femur was larger in than that in the Late Jomon femur, both before and after standardization. In contrast, the area of cortical bone and CI were significantly larger in Initial/Early Jomon femurs than in modern femurs before and after standardization, suggesting that in contrast to the apparent size of femurs, cortical bone may have been larger in Jomon humans. Imamura et al. reported that PBL, the area of cortical bone, and CI did not correlate with age in males, suggesting that the differences in morphological parameters among modern, Initial/Early Jomon, and Late Jomon humans do not reflect differences in the age of the human bones analyzed [9], even though the age of modern bones analyzed by Imamura et al. was biased towards older ages due to their sampling method; therefore, further studies are warranted. Mean cortical bone thickness in each cross-section was also larger in Initial/Early and Late Jomon femurs than in modern femurs, both before and after standardization by diaphyseal length. A previous study reported that the mean thickness of the diaphysis in modern skeletal specimens correlated with the stresses and strains applied to the diaphysis during a one-legged stance [11]. Furthermore, mean thickness values were high in Jomon humans, indicating that their femurs were highly resistant to force, particularly that applied along the bone axis. In previous studies on Jomon humans, PBL corrected by femur length was considered to represent the sturdiness of the femur [31]. This index increased from the middle Jomon Period, and the femur appeared to have become sturdier during this period [32]. In the present study, this index was lower in the Jomon Period, whereas the area and thickness of cortical bone were larger. These results strongly suggest that the femur was sturdier in the Jomon Period, particularly the Late Jomon Period; however, the mechanisms responsible were different.

Humans in the Jomon Period were found to have a more developed linea aspera than modern humans, which may reflect the genetic characteristics or living environment of Jomon humans. It is often expressed as the cross-sectional index, which is the ratio of the long to short diameter of the femoral diaphysis [33]. The index increased with age in Jomon humans and is significantly larger than that of modern humans, who show a smaller degree of increase after puberty, suggesting that it is strongly related to behavior for food gathering [34]. In the present study, instead of this ratio, we used CT to directly measure cortical bone thickness, which enabled us to remove the influence of medullary cavity morphology. The maximum thickness of cortical bone, which is considered to reflect the development of the linea aspera at the center of the diaphysis, was not only significantly different between modern and Jomon humans after standardization by the length of the diaphysis but was also significantly different between Initial/Early and Late Jomon humans. The linea aspera is an attachment site for the adductor muscle, and its degree of development is considered to reflect the extent of movement [35]. Since the linea aspera was significantly developed in femurs of Late Jomon humans, a wide range of movement appeared to be necessary for life in this time period. This is consistent with previous findings showing that the linea aspera of the femur of an Initial Jomon man excavated from Futsukaichi cave (Futsukaichi city, Oita Prefecture, Japan), which is in the vicinity of Hegi cave, was not developed [36]. The period from 4,000 to 4,500 years ago, which was defined as the Late Jomon Period in the present study, was a time of decreasing temperatures in the Holocene world [21]. This was a time when it became difficult to obtain food, and many settlements in other parts of Japan were reported to have disappeared [20]. On the other hand, it was also a time when the sea level was higher than in the Initial/Early Jomon Period and coastline was within approximately 7 kilometers of Hegi cave, in which the Jomon human remains analyzed were found. Freshwater shellfish, such as river snails, have been found in the strata at which Initial/Early Jomon humans were found, while brackish water shellfish, including clams, were detected in the strata at which Late Jomon humans were discovered [18]. This suggests that by the Late Jomon period, the coastline was close enough for people to travel to food gathering from the Hegi caves. This may have contributed to the expansion of the range of activities during this period. The degree of development of the linea aspera may reflect these changes in the living environment. The weight of the archaic man may be estimated from the diameter of the femoral head and the height from the length of the femur. We measured the femoral head diameter and diaphyseal length of all bones used in the present study and examined their relationships. No correlations were found in modern humans, whereas correlations were noted in Initial/Early and Late Jomon humans (*r* = 0.63 and 0.99, respectively). The correlations observed in Late Jomon humans are of interest because they suggest that humans had a uniform body shape that was never obese due to the scarcity of food; however, the small sample size examined may be a factor. The mean values for many of the diaphyseal cross-sectional morphologies were grouped in the order of the modern, Initial/Early Jomon, and Late Jomon populations, and the distribution of points in the principal component analysis was also grouped in this order. In terms of the forward curvature, the mean values for the modern and Initial/Early Jomon populations were similar, while the mean value for Late Jomon population was significantly higher. This suggests that these indices are factors that are influenced by the same living environment, and these data provide an important basis for future comparisons with other populations.

In terms of the lateral curvature, modern and Jomon femurs showed very different patterns. The pattern of curvature was consistent with the axis connecting the center points of the cross-sections at levels 1 and 9, with some showing the primary curvature with the center point of the other level consistently on the outside or inside, and some showing the S-shaped curvature with respect to the axis, with the outside at the top and the inside at the bottom or the inside at the top and the outside at the bottom. In modern humans, 23 out of the 46 femurs measured showed the primary curvature, while those that showed the S-shaped curvature all curved laterally at the top. In contrast, the Initial/Early and Late Jomon populations both showed a more S-shaped curvature and the pattern was mostly medial at the top. In terms of the average degree of curvature, modern humans showed the lateral curvature at the top and almost along the axis at the bottom. A columnar material generally undergoes strain when loaded along its axis. Euler's buckling theory is a qualitative and quantitative description of the form and magnitude of strain [37]. The hip joint has a degree of freedom of lateral rotation, while the knee joint has a fixed degree of freedom of lateral rotation. Applying this combination of degrees of freedom at the hip and knee joints to the theory predicts lateral curvature at the top region and alignment with the bone axis at the bottom region. The results of the present measurements were in good agreement with this prediction. In the case of the anterior curvature, both the hip and knee joints have a degree of freedom in the direction of forward rotation; therefore, the center of the diaphysis is the apex of the curvature, a result that is consistent with this theory. On the other hand, the S-shaped curvature occurs when the hip or knee joint is fixed, and the other joint has a degree of freedom in the lateral direction. If the upper part of the body is flexed laterally, the hip joint may have a lateral degree of freedom or the knee joint may have a degree of freedom in the medial direction. If the upper part of the body is flexing medial at the top, it must have freedom in the opposite direction. The hip joint may tilt laterally when loaded, possibly due to Trendelenburg's sign of abductor weakness. Although the femurs excavated from Hegi cave showed no morphological feature to suggest this pathological condition, an imbalance between the abductor and adductor muscles may cause lateral thrust in the hip joint. In knee osteoarthritis, the knee moves laterally in the case of a medial deformity and medially in the case of a lateral deformity [38]. This lateral and medial thrust may explain the difference in S-shaped patterns between modern and Jomon femurs. Previous studies on European descendants reported that the incidence and types of knee joint diseases were influenced by the historical time [39, 40]. The frequency of patellofemoral osteoarthritis was found to be higher in Asian hunter-gatherers [41]. These findings indicate differences in lifestyle between the modern and Jomon populations that affected the curvature of the femur through differences in the incidence of knee joint diseases. The present results suggest that Jomon humans had more developed adductor muscles than modern humans; however, further investigations on the abductor muscles and the state of the knee joint using other markers are needed. Moreover, a biomechanical analysis using the finite element method is warranted to examine how this difference in the lateral curvature pattern contributed to the robustness of the Jomon Period femur or whether it was a pathological condition.

## 5. Conclusion

The method used in the present study to quantify the morphological characteristics of cortical bone in the femur diaphysis based on CT images revealed marked differences between modern and Jomon Period bones excavated from Hegi cave. The results obtained showed not only differences between the modern and Jomon populations but also among the Initial/Early and Late Jomon Periods according to age. Some of the indices measured exhibited different patterns between the modern and Jomon populations, while others were similar between the modern and Initial/Early Jomon Period populations, but different from the Late Jomon Period population. These differences strongly suggest the influence of different mechanisms on the morphological characteristics of each femur. Climate change and marine transgression during the Late Holocene are strong candidates for explaining the differences observed between the Initial/Early and Late Jomon Period populations. The further application of this method to different paleoanthropological populations is expected to yield novel insights.

## Figures and Tables

**Table 1:** Conventional (years before present, BP) and calibrated radiocarbon ages (calibrated years before present, cal BP) of the femurs excavated from Hegi cave.

No.	Period	Conventional ^14^C age (BP)	AMS lab ID	Calibrated ^14^C age (cal BP) 68.3%	Calibrated ^14^C age (cal BP) 95.4%
1	Late Jomon	3928 ± 28	TKA-18741	4419 (46.0%) 4352 4329 (22.3%) 4298	4507 (2.2%)4490 4438 (88.2%) 4286 4275 (5.1%) 4248
2	Late Jomon	3757 ± 23	PLD-19678	4217 (3.3%) 4211 4153 (65.0%) 4086	4231 (10.8%) 4200 4180 (1.4%) 4169 4159 (70.0%) 4077 4039 (13.2%) 3992
3	Late Jomon	3807 ± 23	PLD-19679	4236 (36.6%) 4194 4188 (31.6%) 4152	4289 (3.4%) 4268 4255 (81.4%) 4142 4126 (10.6%) 4093
4	Late Jomon	3913 ± 29	TKA-18742	4414 (43.8%) 4350 4331 (24.4%) 4296	4420 (95.4%) 4245
6	Late Jomon	3890 ± 23	PLD-19680	4405 (39.7%) 4345 4338 (28.5%) 4293	4411 (95.4%) 4246
16	Early Jomon	5925 ± 25	PLD-18261	6787 (53.0%) 6730 6698 (15.2%) 6679	6830 (1.9%) 6820 6796 (93.6%) 6669
19	Early Jomon	6285 ± 31	TKA-18746	7256 (55.6%) 7192 7180 (12.6%) 7166	7280 (95.0%) 7156 7091 (0.4%) 7082
29	Early Jomon	6286 ± 33	TKA-18753	7256 (22.7%) 7231 7225 (31.1%) 7190 7183 (14.5%) 7166	7307 (1.0%) 7291 7285 (94.0%) 7156 7091 (0.5%) 7082
35	Early Jomon	6667 ± 33	TKA-18757	7578 (22.5%) 7557 7545 (45.7%) 7508	7603 (0.4%) 7600 7593 (93.4%) 7472 7443 (1.6%) 7434
39	Early Jomon	6524 ± 32	TKA-18760	7480 (56.8%) 7421 7380 (11.5%) 7361	7556 (1.6%) 7547 7508 (69.2%) 7418 7390 (24.7%) 7331
40	Early Jomon	6382 ± 31	TKA-18761	7411 (6.4%) 7402 7325 (61.9%) 7264	7421 (17.7%) 7381 7359 (73.6%) 7255 7197 (4.1%) 7177
46	Early Jomon	5864 ± 39	TKA-18766	6738 (68.3%) 6648	6786 (90.3%) 6600 6590 (5.1%) 6561
56	Initial Jomon	8926 ± 30	PLD-18263	10183 (31.3%) 10120 10064 (13.4%) 10037 10028 (7.4%) 10010 9991 (16.2%) 9960	10193 (35.2%) 10110 10080 (60.3%) 9909
59	Initial Jomon	8564 ± 51	MTC-15609	9550 (68.3%) 9488	9664 (3.1%) 9640 9631 (92.4%) 9470
60	Early Jomon	6514 ± 39	TKA-18770	7473 (21.1%) 7444 7434 (14.2%) 7420 7383 (33.0%) 7335	7554 (0.8%) 7549 7507 (51.6%) 7412 7402 (43.0%) 7325

**Table 2:** Eigenvectors of a main component analysis with morphological parameters normalized with femoral lengths in modern, Initial/Early Jomon, and Late Jomon Period populations. These vectors were used to calculate the first and second principal component scores (*Z*1 and *Z*2), shown in Figure 6, respectively.

	*Z*1	*Z*2
Area of cortical bone	0.571	-0.003
Cross-sectional area	0.552	-0.202
Cortical index	-0.056	0.616
Periosteal border length	0.517	-0.133
Mean cortical bone thickness	0.292	0.544
Maximum cortical bone thickness	0.111	0.517

**Table 3:** Types of femoral curvatures. The proportions of each type of curvature significantly differed among modern, Initial/Early, and Late Jomon Period populations.

	Primary curvature		S-shaped curvature	
	Lateral	Medial	Lateral-medial	Medial-lateral
Modern	19	4	23	0
Initial/Early Jomon	0	3	0	7
Late Jomon	0	0	1	4

## Data Availability

Previously reported morphological data of modern Japanese people were used to support this study and are available at doi:10.1111/joa.13399 and doi:10.1111/joa.13060. These prior studies are cited at relevant places within the text as references [9, 10].
